# Hamiltonian energy in a modified Hindmarsh–Rose model

**DOI:** 10.3389/fnetp.2024.1362778

**Published:** 2024-03-26

**Authors:** Qianqian Zheng, Yong Xu, Jianwei Shen

**Affiliations:** ^1^ School of Science, Xuchang University, Xuchang, Henan, China; ^2^ School of Mathematics and Statistics, Northwestern Polytechnical University, Xi’an, Shaanxi, China; ^3^ School of Mathematics and Statistics, North China University of Water Resources and Electric Power, Zhengzhou, Henan, China

**Keywords:** HR, pattern formation, network, matrix, Turing instability, delay

## Abstract

This paper investigates the Hamiltonian energy of a modified Hindmarsh–Rose (HR) model to observe its effect on short-term memory. A Hamiltonian energy function and its variable function are given in the reduced system with a single node according to Helmholtz’s theorem. We consider the role of the coupling strength and the links between neurons in the pattern formation to show that the coupling and cooperative neurons are necessary for generating the fire or a clear short-term memory when all the neurons are in sync. Then, we consider the effect of the degree and external stimulus from other neurons on the emergence and disappearance of short-term memory, which illustrates that generating short-term memory requires much energy, and the coupling strength could further reduce energy consumption. Finally, the dynamical mechanisms of the generation of short-term memory are concluded.

## 1 Introduction

Short-term memory is a primary cognitive function of the brain. The transitions between the spontaneous and persistent states could lead to the emergence and disappearance of short-term memory in a bistable system (Amit and Brunel, 1997). Continuous neural activity without external inputs was deemed an expression of short-term memory (Wang, 2001). A phenomenological model of spatial working memory was developed to examine the dynamical interactions of multiple feedback mechanisms (Carter and Wang, 2007). A growing body of evidence suggests memories may be kept through the mutual effect of persistent neural activity and activity-silent dynamics (Stokes, 2015; Barbosa et al., 2020). Gaussian noise was treated as an essential factor in neuronal activity and its toggle switch (memory maintenance) (Zheng et al., 2020b). Memory maintenance through persistent neural activity and a synaptic mechanism was compared in mice and two types of artificial neural networks to show their differences (Hu et al., 2021). Then, computational modeling was constructed to prove how the circuits and networks affect working memory, which provides a novel theory for memory maintenance (Ghazizadeh and Ching, 2020). In addition, the encoding style of the input information of the short-term memory was investigated to illustrate the dynamical mechanisms of short-term memory (Ichikawa and Kaneko, 2021; Jones and Ching, 2022; Zhou et al., 2023). Hamilton energy, representing the utilization of energy (actual energy in the generation of short-term memory), should be considered to illustrate the dynamic mechanism of the generation of short-term memory.

Hindmarsh–Rose (HR) model (1) (*x* is the membrane potential, *y* is the recovery variable of the fast current of *K* ^+^ or *Na* ^+^, and *z* is the adaptation variable of the slow current of *Ca* ^+^ or other ions) was proposed to show the membrane potential of neuronal activity (Hindmarsh and Rose, 1982), which has rich dynamical behaviors (Wang et al. 2021a; Wang et al. 2021b; Wang et al. 2022).

$$\begin{array}{ll} \frac{dx}{dt} & =y-ax^{3}+bx^{2}+I_{\mathrm{ext}}-z,\\ \frac{dy}{dt} & =c-dx^{2}-y,\\ \frac{dz}{dt} & =r\left(s\left(x-x_{r}\right)-z\right). \end{array}$$ (1)

The synchronization and bifurcation (Song and Xu, 2013; Liebovitch et al., 2011; Kumar et al., 2016; Zheng et al. 2020a; Zheng et al. 2024; Zheng et al. 2023) of the HR model were often studied to demonstrate the dynamical mechanism of chaotic bursting or spikes (Shi and Wang, 2012; Wu et al., 2016; You, 2023a). The interplay between neurons was analyzed to present the effect of the parameters and coupling strength on the appropriate functioning of the system (Lepek and Fronczak, 2018; Rajagopal et al., 2019; You, 2023b). Energy is necessary for neuron activity (Attwell and Laughlin, 2001). The Hamiltonian energy function is a vital tool to evaluate energy consumption when neurons are active (Torrealdea et al., 2006). The average energy consumption of the HR model was given to display the energy consumption ratio in different situations, which could help optimize energy use (Torrealdea et al., 2009; Song et al., 2015; Usha and Subha, 2019). The Hamilton energy balance of different functional neurons was discussed through the coupling strength, which contributes to designing functional assistive devices (Zhang et al., 2022; Yu et al., 2023). Although the HR model could explain the generation of short-term memory (Zheng et al., 2022), the utilization of energy should be further stated in short-term memory.

Short-term memory results from neuronal activity coming with a change in energy, and a physical neuron circuit plays a vital role in the synergistic effect of neurons and the generation of short-term memory. In this paper, the pattern formation of a modified HR model is investigated to find the dynamical mechanism of how the coupling strength and links (degree) affect the generation of short-term memory. The Hamiltonian energy function is derived in the HR model with a single node, which means the energy consumption varies at different states of neuronal activity. Then, the degree and stimuli from other neurons are studied through bifurcation, which means the energy is necessary to generate short-term memory. Finally, the related dynamical and biological mechanisms are obtained.

## 2 Model description

As the membrane potential of neurons is often coupled with others, the following network-organized HR model is introduced:

$$\begin{array}{ll} \frac{dx_{i}}{dt} & =y_{i}-ax_{i}^{3}+bx_{i}^{2}+I_{\mathrm{ext}}-z_{i}+d_{1}\overset{n}{\underset{j=1}{\sum }}L_{ij}\left(t\right)x_{j},\\ \frac{dy_{i}}{dt} & =c-dx_{i}^{2}-y_{i},\frac{dz_{i}}{dt}=r\left(s\left(x_{i}-x_{r}\right)-z_{i}\right), \end{array}$$ (2)

where *x*_*i*_ is the membrane potential, *I* represents the *ith* neuron and *i* = 1, … , *n*, *y*_*i*_ is the recovery variable of the fast current of *K* ^+^ or *Na* ^+^, and *z*_*i*_ is the adaptation variable of the slow current of *Ca* ^+^ or other ions. *D*_1_ is the coupling strength between neurons. *L*_*ij*_(*t*) = *A*_*ij*_ – *δ*_*ij*_ *k*_*i*_, where *A*_*ij*_ is the adjacent matrix and *k*_*i*_ is the degree of the *ith* node.

In order to obtain the Hamiltonian energy function of system (2), we consider a simplified model with a single node through the mean-field approach (McCullen and Wagenknecht, 2016). The reduced system is

$$\begin{array}{c} \frac{dx_{i}}{dt}=y_{i}-ax_{i}^{3}+bx_{i}^{2}+I_{\mathrm{ext}}-z_{i}+d_{1}k_{i}\left(x_{0}-x_{i}\right),\\ \frac{dy_{i}}{dt}=c-dx_{i}^{2}-y_{i},\\ \frac{dz_{i}}{dt}=r\left(s\left(x_{i}-x_{r}\right)-z_{i}\right), \end{array}$$ (3)

where (*x*_0_, *y*_0_, *z*_0_) (Zheng et al., 2022) is the equilibrium point of system (2) without a network, and *x*_0_ makes *I*_*ext*_ = *f*(*x*) = −*c* + *dx* ^2^ + *ax* ^3^ − *bx* ^2^ + *s* (*x* − *x*_*r*_) + *I*_*ext*_ + *d*_1_ *k*_*i*_ (*x*_0_ − *x*_*i*_) hold. In addition, *x*_0_ is the external stimulus from other neurons.

In this paper, we mainly investigate the dynamical behaviors of system (3) and its Hamiltonian equation. According to Helmholtz’s theorem (Donald and Rose, 1986), an autonomous ordinary differential equation $\dot{X}=F\left(X\right)$ (*F*(*X*) can be treated as the velocity vector field) can be described in the usual forms of a Hamiltonian equation:

$$\left.\begin{array}{c} \dot{X}=G\left(X\right)\nabla H\left(X\right), \end{array}\right.$$

where *G*(*X*) is a skew-symmetric matrix in the Hamiltonian system. If *G*(*X*) is not a skew-symmetric matrix in a generalized Hamiltonian system, *G*(*X*) can be divided into two parts *G*(*X*) = *G*_1_(*X*) + *G*_2_(*X*): a skew-symmetric matrix *G*_1_(*X*) and a symmetric matrix *G*_2_(*X*) (Sarasola et al., 2004). *H*(*X*) is an energy function. Then, we have

$$\left.\begin{array}{c} \dot{X}=\left(G_{1}\left(X\right)+G_{2}\left(X\right)\right)\nabla H\left(X\right). \end{array}\right.$$

For the network-organized HR model (3), it can be written as (Torrealdea et al., 2006; Torrealdea et al., 2009; Song et al., 2015; Usha and Subha, 2019)

$$\left(\begin{array}{c} {\dot{x}}_{i}\\ {\dot{y}}_{i}\\ {\dot{z}}_{i} \end{array}\right)=\left.\begin{array}{c} \left(G_{1}\left(x_{i},y_{i},z_{i}\right)+G_{2}\left(x_{i},y_{i},z_{i}\right)\right)\nabla H\left(x_{i},y_{i},z_{i}\right), \end{array}\right.$$

where

$$\left.\begin{array}{c} F_{1}\left(x_{i},y_{i},z_{i}\right)=G_{1}\left(x_{i},y_{i},z_{i}\right)\nabla H\left(x_{i},y_{i},z_{i}\right) \end{array}\right.=\left(\begin{array}{c} y_{i}-z_{i}+d_{1}k_{i}\left(x_{0}-x_{i}\right)\\ -dx_{i}^{2}\\ rsx_{i} \end{array}\right),$$

$$\left.\begin{array}{c} F_{2}\left(x_{i},y_{i},z_{i}\right)=G_{2}\left(x_{i},y_{i},z_{i}\right)\nabla H\left(x_{i},y_{i},z_{i}\right) \end{array}\right.=\left(\begin{array}{c} -ax_{i}^{3}+bx_{i}^{2}+I_{\mathrm{ext}}-k_{i}x_{i}\\ c-y_{i}\\ -rsx_{r}-rz_{i} \end{array}\right).$$

Furthermore, we obtain the Hamiltonian energy function as (Zhang et al., 2022)

$$\left\{\begin{array}{c} \nabla H{\left(x_{i},y_{i},z_{i}\right)}^{T}F_{1}\left(x_{i},y_{i},z_{i}\right)=0,\;\\ \dot{H}\left(x_{i},y_{i},z_{i}\right)=\nabla H{\left(x_{i},y_{i},z_{i}\right)}^{T}F_{2}\left(x_{i},y_{i},z_{i}\right).\; \end{array}\right.$$

Namely,

$$\left.\begin{array}{c} \left(y_{i}-z_{i}+d_{1}k_{i}\left(x_{0}-x_{i}\right)\right)\frac{\partial H\left(x_{i},y_{i},z_{i}\right)}{\partial x_{i}}-dx_{i}^{2}\frac{\partial H\left(x_{i},y_{i},z_{i}\right)}{\partial y_{i}}+rsx_{i}\frac{\partial H\left(x_{i},y_{i},z_{i}\right)}{\partial z_{i}}=0, \end{array}\right.$$

where a general solution can be expressed as

$$\left.\begin{array}{c} H\left(x_{i},y_{i},z_{i}\right)={\left(y_{i}-z_{i}+d_{1}k_{i}\left(x_{0}-x_{i}\right)\right)}^{2}+\frac{2}{3}dx_{i}^{3}+rsx_{i}^{2}, \end{array}\right.$$

and

$$\left.\begin{array}{c} H_{t}=\dot{H}\left(x_{i},y_{i},z_{i}\right)=\left(2rsx_{r}+2rz_{i}+2c-2y_{i}\right)\left(d_{1}k_{i}\left(x_{0}-x_{i}\right)\right)\\ -2adx_{i}^{5}+\left(-2ars+2bd\right)x_{i}^{4}+\left(2brs-2dk_{i}\right)x_{i}^{3}+\left(-2k_{i}rs+2I_{\mathrm{ext}}d\right)x_{i}^{2}+2I_{\mathrm{ext}}rsx_{i}+2rsx_{r}y-2rsx_{r}z_{i}+2ry_{i}z_{i}-2rz_{i}^{2}+2cy_{i}-2cz_{i}-2y_{i}^{2}+2y_{i}z_{i}. \end{array}\right.$$

## 3 Numerical results and discussion

**FIGURE 1:**
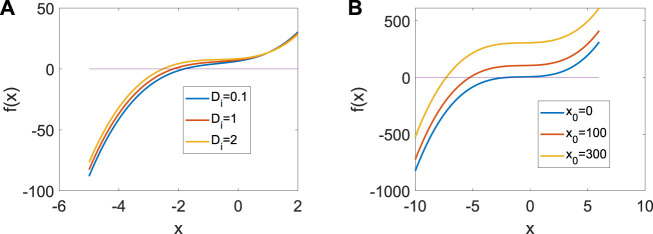
Distribution of the equilibrium point in system (3) when *a* = | 1, *b* = | 3, *c* = | 1, *d* = | 5, *r* = | 0.01, s = | 4. **(A)** Distribution of the equilibrium point when *x*_0_ | = | 1. **(B)** Distribution of the equilibrium point when *D*_*i*_ = | 1.

In this section, the finite difference method is applied to find numerical solutions for the network-organized HR model (3) with time step *dt* = 0.01. These parameters *a* = 1, *b* = 3, *c* = 1, *d* = 5, *r* = 0.01, s = 4 are set (Zheng et al., 2022). The small-world network is constructed with *W* (*n*, *K*, *p*) (the number of node *n*, nearest neighbor *K*, and reconnection probability *p*), which can be found can be found at https://github.com/zhengqianqian35/network-code. We give the concept of the average Hamiltonian energy for time and nodes:

$$\left.\begin{array}{c} H_{1}=\frac{\int_{t_{0}}^{t_{0}+T}H\left(x_{i},y_{i},z_{i}\right)dt}{T}, \end{array}\right.$$

$$\left.\begin{array}{c} H_{2}=\left\{max\left(\dot{H}\left(x_{i},y_{i},z_{i}\right)\right),min\left(\dot{H}\left(x_{i},y_{i},z_{i}\right)\right)\right\}, \end{array}\right.$$

where the integration period is set at *T* = 5,000 time units. In order to exclude the influence of initial conditions, *t*_0_ is the starting time of the cycle after the system tends to a stable state. In addition, we assume *H* = *H* (*x*_*i*_, *y*_*i*_, *z*_*i*_)/100. From Figure 1, only one real equilibrium point (*x*_0_, *y*_0_, *z*_0_) exists in system (1) when *a* = 1, *b* = 3, *c* = 1, *d* = 5, *r* = 0.01, s = 4, which guarantees the uniqueness of system (3).

**FIGURE 2:**
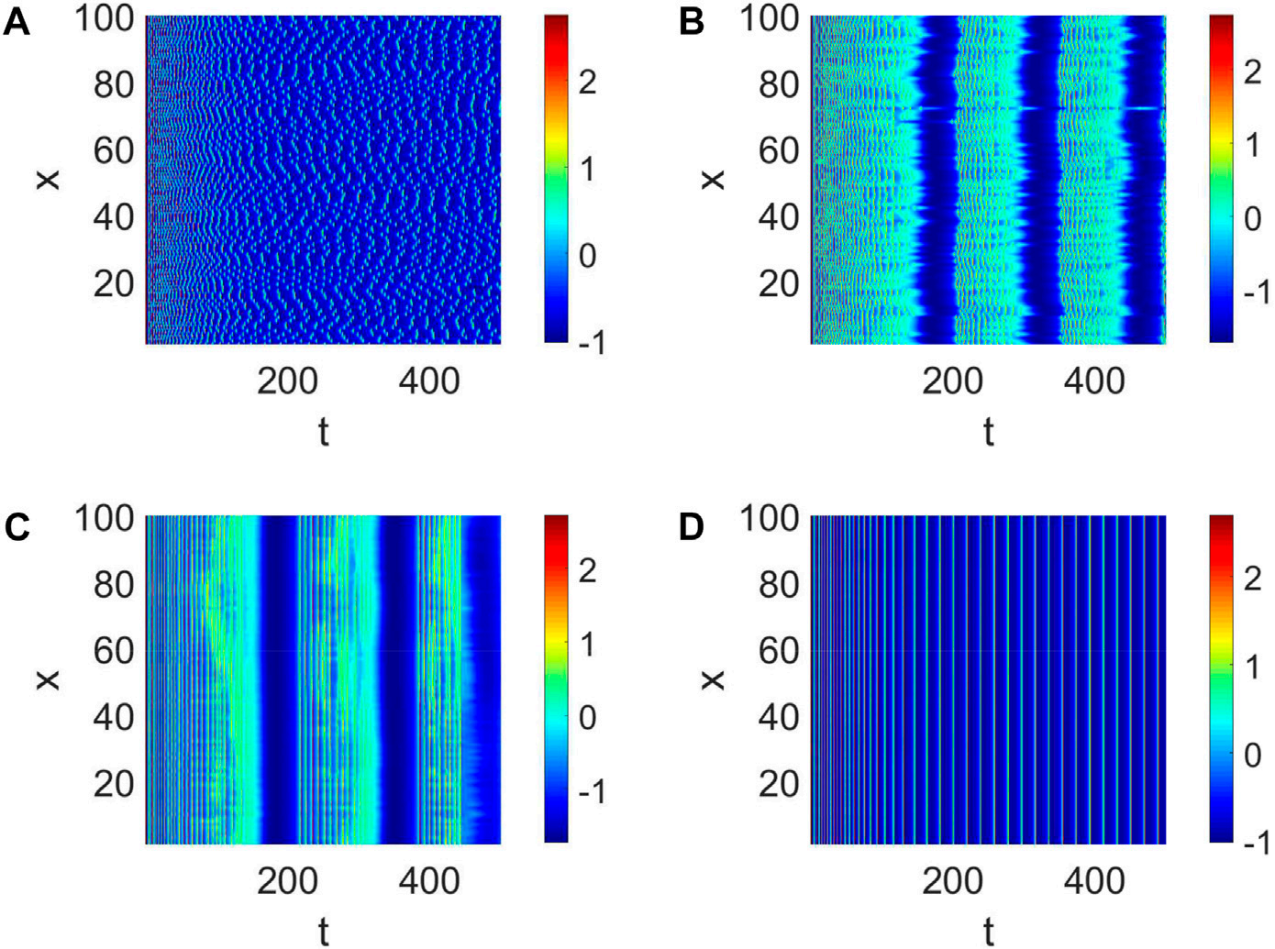
Pattern formation when *I*_*ext*_ = | 4 and *W* (100, | 8, | 0.01). **(A)** Pattern formation when *d*_1_ | = | 0.01. **(B)** Pattern formation when *d*_1_ | = | 0.05. **(C)** Pattern formation when *d*_1_ | = | 0.3. **(D)** Pattern formation when *d*_1_ | = | 1.

In general, the generation of neural function results from the collaboration of multiple neurons. Therefore, we consider the strength *d*_1_ of the coupling between neurons and the number of links *K*. First, a small-world network with *W* (100, 8, 0.01) is given. The pattern formation is chaotic (Figure 2A) when the strength *d* = 0.01 is weak, which means the nervous system does not work. The pattern formation starts to become clear and tends to sync with the increase of *d*_1_ (Figure 2b,c). Ultimately, the pattern formation becomes synchronized; namely, all the neurons become completely phase synchronized (Figure 2D). The short-term memory needs to be clarified when *d*_1_ is weak. Only when all the neurons work perfectly together is a clear short-term memory formed (Figure 2), which is also the mechanism by which adequate short-term memory is produced.

**FIGURE 3:**
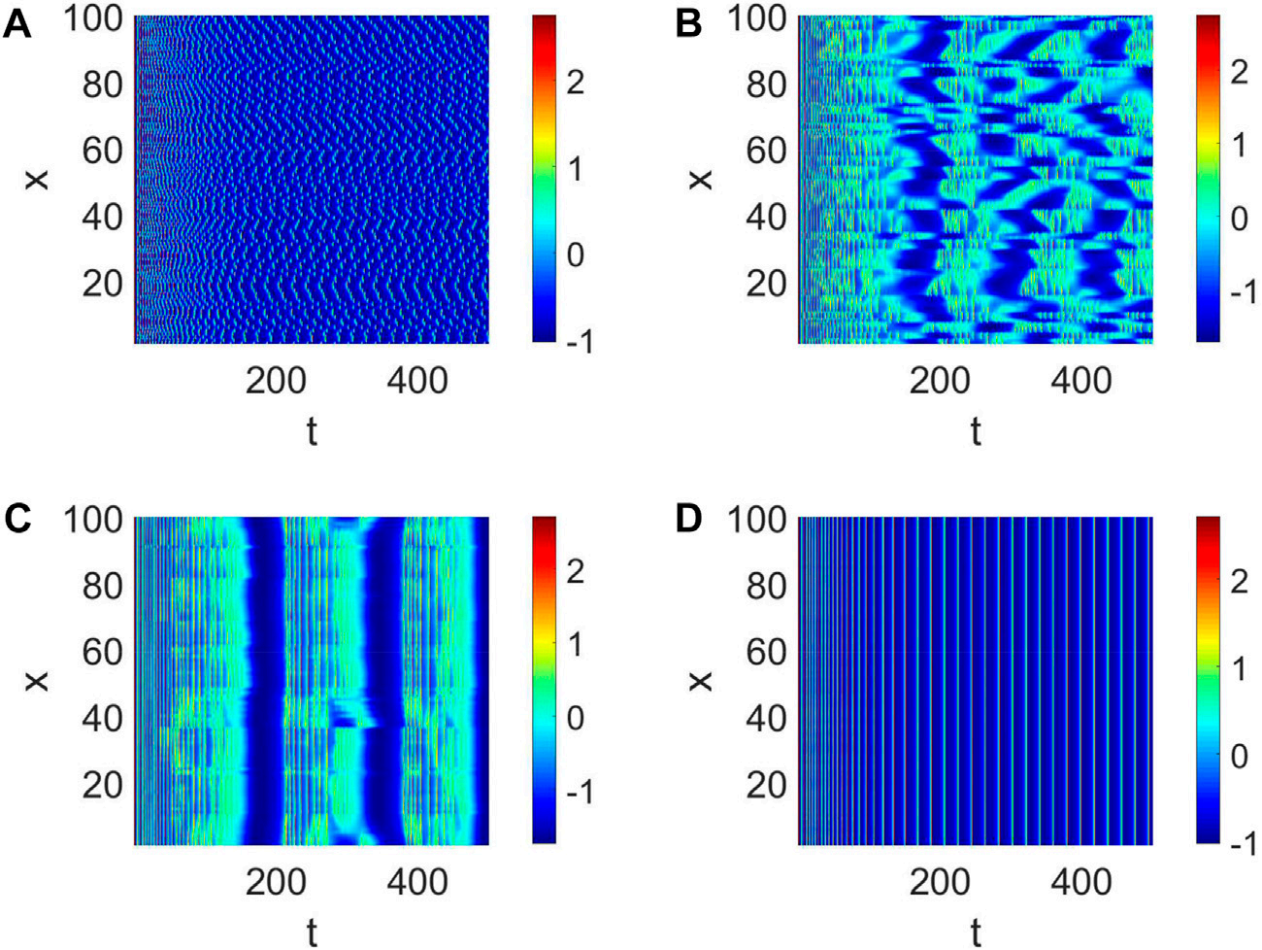
Pattern formation when *I*_*ext*_ = | 4, *d*_1_ | = | 1 and *W* (100, *K*, | 0.01). **(A)** Pattern formation when *K* = | 0. **(B)** Pattern formation when *K* = | 2. **(C)** Pattern formation when *K* = | 4. **(D)** Pattern formation when *K* = | 6.

Then, the number of cooperative neurons will be considered to generate short-term memory. The links between neurons can be treated as the number of collaborative neurons in our analysis, which could be measured by *K*. When *K* is small, the pattern formation is chaotic, and every neuron is relatively independent (Figure 3A). This condition is not suitable for the generation of short-term memory. When *K* = 2, the pattern formation shows some neurons are in sync (Figure 3B); namely, multiple short-term memories are produced simultaneously. In this case, short-term memory is often fuzzy. The short-term memory gradually becomes clear when *K* becomes large (Figure 3C). Eventually, multiple neurons work together to form a clear short-term memory when all the neurons are in sync (Figure 3D).

Finally, it is found that the link probability does not work because *p* cannot change the number of cooperative neurons and the coupling strength.

### 3.1 Hamiltonian energy with external stimulus

System (3) can be written as

$$\left.\begin{array}{c} \frac{dx_{i}}{dt}=y_{i}-x_{i}^{3}+3x_{i}^{2}+I_{\mathrm{ext}}-z_{i}+D_{i}\left(x_{0}-x_{i}\right),\\ \frac{dy_{i}}{dt}=1-5x_{i}^{2}-y_{i},\\ \frac{dz_{i}}{dt}=0.04x_{i}-z_{i}+0.064, \end{array}\right.$$ (4)

where *D*_*i*_ = *d*_1_ *k*_*i*_ (*d*_1_ = 0.01), and the effect of *x*_0_ is similar to *I*_*ext*_ when *D*_*i*_ is a constant. *I*_*ext*_ and the coupling strength play a vital role in the electrical activity, which is the basis of the generation of fire. Therefore, we consider the role of *I*_*ext*_ and *D*_*i*_ in the Hamiltonian energy, change in Hamiltonian energy, and membrane potential when *x*_0_ = 1.

**FIGURE 4:**
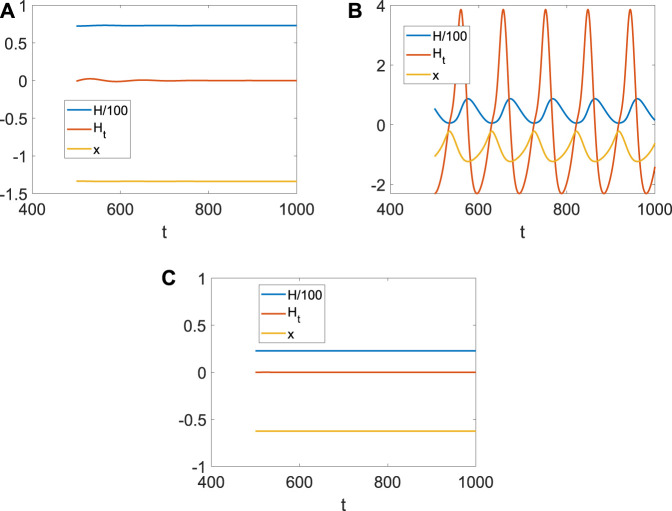
Hamiltonian energy, change in Hamiltonian energy, and membrane potential when *I*_*ext*_ = | 1, *x*_0_ | = | 1. **(A)** Evolution when *D*_*i*_ = | 0.1. **(B)** Evolution when *D*_*i*_ = | 1. **(C)** Evolution when *D*_*i*_ = | 1.5.

It is well known that the coupling between neurons is necessary for generating fire or short-term memory (Figure 2). Because only the *ith* neuron evolutes with system (4), and other neurons are fixed at (*x*_0_, *y*_0_, *z*_0_), *D*_*i*_ can also be regarded as the size of the network degree. When the coupling strength is small, or the number of links is few, no spike or memory is generated; namely, the neurons are resting (Figure 4A). The membrane potential began to change periodically with the increase in *D*_*i*_, which means the emergence and disappearance of short-term memory (Figure 4B). Meanwhile, the Hamiltonian energy and change in Hamiltonian energy change with the membrane potential, which means the generation of the short-term memory takes more energy. However, short-term memory is the result of multiple neurons working together. If one neuron is very tightly connected to other neurons, all the neurons will tend to be in one state because other neurons are fixed at (*x*_0_, *y*_0_, *z*_0_). Namely, system (4) of a neuron will tend to a stable state when *D*_*i*_ is larger (Figure 4C).

**FIGURE 5:**
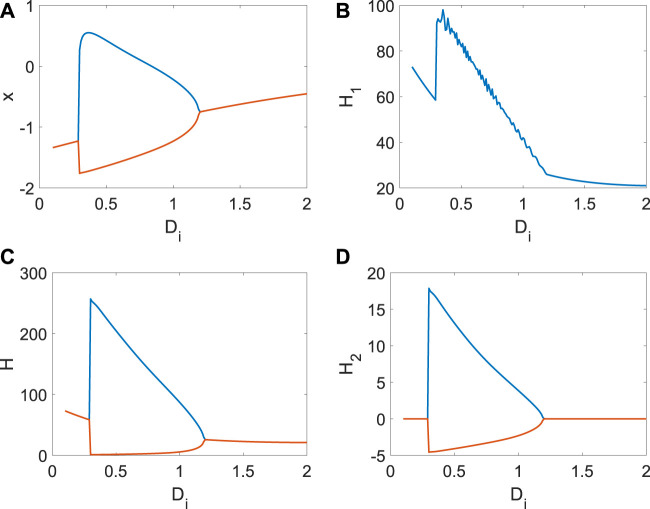
Average Hamiltonian energy, average change in Hamiltonian energy, and membrane potential when *I*_*ext*_ = | 1. **(A)** Bifurcation of membrane potential. **(B)** Average Hamiltonian energy. **(C)** Max–min value of Hamiltonian energy. **(D)** Max–min value of Hamiltonian energy variation.

Next, we show the continuous changes in *x*, *H*_1_, *H*_2_ with *D*_*i*_ (Figure 5). From Figure 5A, the bifurcation occurs with the increase in *D*_*i*_. The average Hamiltonian energy decreases gradually at the beginning because the utilization of energy is relatively low when the neuron is in a resting state (Figure 5B). When the membrane potential is periodic, the average Hamiltonian energy will be a sudden increase (Figure 5B). Meanwhile, the rise of coupling strength also reduces the consumption of Hamiltonian energy, which is why the average Hamiltonian energy decreases with *D*_*i*_ (Figure 5B). It is found that the max–min value of Hamiltonian energy (Figure 5C) and its variation (Figure 5D) is significantly associated with the bifurcation, which is essential to show the relationship between the consumption of energy and the membrane potential (the generation of short-term memory). In a word, generating short-term memory will take a lot of energy, and the coupling strength could further reduce energy consumption.

**FIGURE 6:**
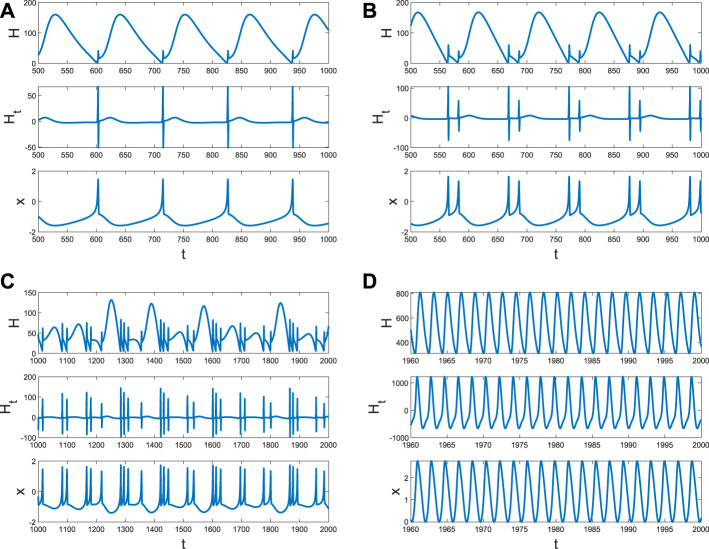
Hamiltonian energy, change in Hamiltonian energy, and membrane potential when *D*_*i*_ = | 0.1, *I*_*ext*_ = | 1. **(A)** Evolution when *x*_0_ | = | 5. **(B)** Evolution when *x*_0_ | = | 10. **(C)** Evolution when *x*_0_ | = | 20. **(D)** Evolution when *x*_0_ | = | 200.

**FIGURE 7:**
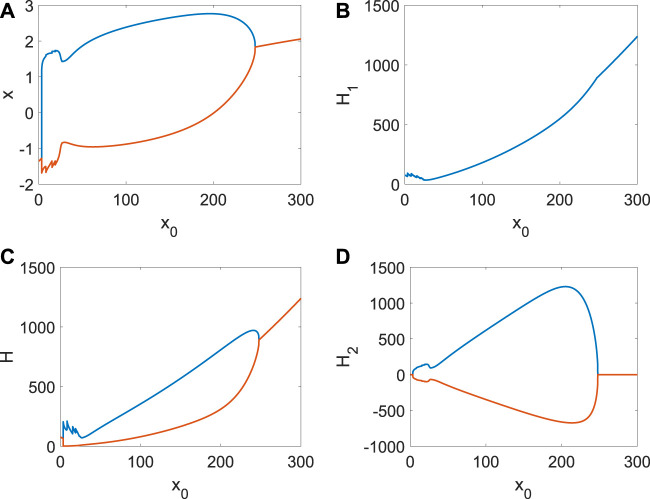
Average Hamiltonian energy, change in Hamiltonian energy, and membrane potential when *D*_*i*_ = | 1, *I*_*ext*_ = | 1. **(A)** Bifurcation of membrane potential. **(B)** Average Hamiltonian energy. **(C)** Max–min value of Hamiltonian energy. **(D)** Max–min value of Hamiltonian energy variation.

The role of *x*_0_ from other neurons’ external stimulus (Li et al., 2024b; Li et al., 2024a; Du et al., 2024) is the same as *I*_*ext*_ when other parameters are fixed. When the external stimulation of other neurons contributing to the *ith* neuron is weak, system (4) (the Hamiltonian energy, change in Hamiltonian energy, and membrane potential) is stable (Figure 4A). The periodical spike occurs (Figure 6A) in the membrane potential when *x*_0_ increases, which corresponds to the emergence and disappearance of short-term memory. It is found that energy consumption is relatively large in preparation for the spike, and the energy varies significantly in the spike (Figure 6A), which can be treated as an indicator of the generation of the spike (short-term memory). The frequency of spikes will increase (Figure 6B) when external stimuli are enhanced. However, it is insufficient to support two identical spikes of membrane potential *x* (Figure 6B) due to the lack of external stimulus or the Hamiltonian energy *H* (Figure 6B). Therefore, the formation of short-term memory requires a process of accumulating energy, and the energy breaks out when a spike occurs. The more energy accumulates, the greater the energy change (*H*_*t*_). If *x*_0_ continues to increase, there will be more spikes, but their intensity is different (Figure 6C). A constant spike is created when *x*_0_ is very large (Figure 6D), which is also the ordinary emergence and disappearance of short-term memory. However, the external stimulus from other neurons will inhibit the generation of the spike and put the *ith* neuron in a resting state (Figure 7A). From Figure 7, the spike is impossible without extensive external energy input, and it has excellent fluctuations at the beginning (Figure 7A). We find the average Hamiltonian energy increases with *x*_0_ (Figure 7B). The max–min value of Hamiltonian energy (Figure 7C) and the max–min value of Hamiltonian energy variation (Figure 7D) are consistent with the bifurcation of *x*.

Finally, we conclude the dynamical mechanism of the generation of short-term memory: the energy from other neurons is necessary for short-term memory, proving that short-term memory results from multiple neuronal activities. Energy requires a process of accumulation to maintain a complete spike. The excessive influence of other neurons can make the *ith* neurons lose their dominance and align with the dynamic behaviors of other neurons.

## 4 Conclusion

Energy plays a vital role in neuronal activity, which is the basis of the generation of short-term memory. In this paper, the pattern formation could represent the collecting dynamics of short-term memory through the Hamiltonian energy, showing the neuronal activity in generating short-term memory. Therefore, the interplay between neurons is considered through a simple network to show the effect of the external stimulus and coupling strength (degree) on the dynamical behaviors. It is found that the Hamiltonian energy, change in the Hamiltonian energy, and membrane potential are consistent. The excessive influence of other neurons can make the *ith* neurons lose their dominance and align with the dynamic behaviors of other neurons, which could show the synergistic effect of neurons through a physical neuron circuit. In addition, the energy from other neurons is necessary for short-term memory, proving that short-term memory results from multiple neuronal activities. Generating short-term memory requires much energy, and energy requires a process of accumulation to maintain a complete spike. Meanwhile, the coupling strength could further reduce energy consumption, which provides a novel way to reduce the energy consumption in information storage and processing. However, more short-term memory descriptions should be completed next.

## Data Availability

The original contributions presented in the study are included in the article/Supplementary Material; further inquiries can be directed to the corresponding authors.
